# Efficacy and predictive factors of endoscopic ultrasound-guided ethanol ablation in benign solid pancreatic tumors

**DOI:** 10.1007/s00464-022-09833-3

**Published:** 2023-04-20

**Authors:** Jin Ho Choi, Woo Hyun Paik, Sang Hyub Lee, Min Woo Lee, In Rae Cho, Ji Kon Ryu, Yong-Tae Kim

**Affiliations:** 1 Department of Medicine, Samsung Medical Center, Sungkyunkwan University, School of Medicine, Seoul, South Korea; 2 Department of Internal Medicine, Liver Research Institute, Seoul National University College of Medicine, Seoul, South Korea

**Keywords:** Endoscopic ultrasound, Ethanol ablation, Pancreatic neuroendocrine tumor, Solid pseudopapillary tumor

## Abstract

**Backgrounds and Objectives:**

Endoscopic ultrasound-guided ethanol ablation (EUS-EA) has recently been introduced for the management of solid pancreatic tumors, including pancreatic neuroendocrine tumors (PNETs) and solid pseudopapillary tumors (SPTs). The study aims to evaluate the efficacy and predictive factors for response of EUS-EA in solid pancreatic tumors.

**Methods:**

Between October 2015 and July 2021, 72 patients who underwent EUS-EA for solid pancreatic tumors were included. The study outcomes were to evaluate the efficacy of EUS-EA with complete remission (CR) and objective response, and their predictive factors.

**Results:**

During follow-up, 47 patients were diagnosed with PNETs and 25 with SPTs. Eight cases reached CR and 48 reached objective response. When compared with SPTs, PNETs showed similar duration to reach CR (median not reached; *p* = 0.319), but shorter duration to reach objective response (PNETs: median 20.6 months, 95%CI 10.26–30.88; SPTs: median 47.7 months, 95%CI 18.14–77.20; *p* = 0.018). Ethanol dosage > 0.35 ml/cm^3^ shortened the duration to reach CR (median not reached; *p* = 0.026) and objective response (median 42.5 months, 95%CI 25.34–59.66 vs. 19.6 months, 95%CI 10.17–29.09; *p* = 0.006). CR had no significant predictive factors, but PNETs showed significant predictive factors for objective response (HR 3.34, 95%CI 1.07–10.43; *p* = 0.038). Twenty-seven patients experienced adverse events, and there were two severe cases.

**Conclusion:**

EUS-EA for pancreatic solid lesions seems feasible as a local treatment for patients who refuse or are unfit for surgery. Additionally, PNETs seem to be the better candidate for EUS-EA.

Various types of solid pancreatic neoplasms have been reported, some of which require invasive treatment [1]. Pancreatic neuroendocrine tumors (PNETs) account for less than 3% of all pancreatic lesions. Conservative management with a wait-and-see strategy for small asymptomatic PNETs seems to be safe [2]. However, surgical resection is still the treatment of choice, especially for symptomatic PNETs or locally invasive PNETs [3]. Solid pseudopapillary neoplasms (SPNs) of the pancreas, which account for 0.9–2.7% of all pancreatic tumors, have generally been regarded as low-grade malignant tumors which preferentially develop in young women and have a good prognosis with surgery [4, 5].

Endoscopic ultrasound-guided ethanol ablation (EUS-EA) is a minimally invasive treatment of pancreatic lesions. Several previous studies have reported promising results for EUS-EA as an alternative treatment to surgical resection [6–10]. This procedure has mostly been used to treat functional PNETs, but the number of patients included in those studies was small and the follow-up duration was relatively short. Recently, a retrospective study of 33 patients has reported a high complete ablation rate of 60% with EUS-guided ethanol-lipiodol ablation for small PNETs [8]. However, there is not enough evidence to define the proper indication and to evaluate long-term efficacy of this ethanol-using ablative therapy for solid pancreatic masses.

In this study, we retrospectively reviewed patients who underwent EUS-EA for solid pancreatic masses to evaluate its effectiveness and to suggest proper indications for this procedure.

## Methods

### Study design and patients

This retrospective cohort study was conducted at a single tertiary center between October 2015 and July 2021. The study was approved by the Institutional Review Board of Seoul National University Hospital, Korea (IRB no. H-2203–023-1303), and the need for informed consent was waived by the IRB.

This study included patients who underwent EUS-EA for solid tumors of the pancreas, were followed-up more than 3 months, underwent follow-up imaging more than once after EUS-EA, and were at least 20 years old. Patients who were unable to undergo planned EUS-EA due to clinical or technical problems, who did not undergo follow-up imaging, or who were diagnosed with non-neoplastic diseases were excluded.

### Procedures and follow-up after the procedure

Patients received EUS-EA from four endo-sonographers (Y. Kim, J. K. Ryu, S. H. Lee, W. H. Paik) who are experts in endoscopic ultrasound (EUS) with an annual average of 125 diagnostic EUS and 160 EUS-guided fine needle aspirations. EUS-EA was performed immediately after tissue acquisition during the same session. A curvilinear-array echoendoscope (GF-UCT2000, GF-UCT 240, GF-UCT 260; Olympus Optical Co, Tokyo, Japan) with a 7.5-MHz transducer (EU-M 2000; Olympus Optical Co, Tokyo, Japan, Aloka Alpha 5 and 10; Hitachi Aloka Medical, Ltd, Tokyo, Japan) was used. EUS-EA was performed through transgastric or transduodenal puncture of the solid pancreatic mass using a 19-, 22- or 25-gauge needle (EchoTip Ultra; Cook Endoscopy, Winston-Salem, NC, EZ Shot 2 or 3™; Olympus Medical, Tokyo, Japan, Expect™; Boston Scientific, MA, USA).

Solid pancreatic masses were treated via using the following protocol: (1) measure the longest diameter, (2) after tissue acquisition for diagnosis, puncture the lesion and gently inject 99% ethanol, (3) inject continuously until hyperechoic “blush” is seen inside the whole mass or resistance is felt. Ethanol injection was performed by moving the intratumoral position of the needle after a single puncture to enable ethanol to reach the entire mass. Injection was stopped upon ethanol spillage.

In general, patients with no major problems after the procedure underwent imaging tests 3–6 months later, such as computed tomography (CT), abdominal ultrasound, and magnetic resonance imaging, and imaging tests were performed every 6–12 months. Further follow-up strategy and treatment were left to the physicians’ discretion taking into consideration the response to treatment and the patients’ wishes.

### Definitions of study events and outcomes

The primary objective of this study was to evaluate the efficacy of EUS-EA for treating solid pancreatic tumors. The evaluation of the response to the procedure was based on Response Evaluation Criteria in Solid Tumors (RECIST) guidelines, with the longest diameter defined as complete remission (CR), partial response (PR), stable disease (SD), and progressive disease (PD) [11]. Objective response was defined as tumor shrinkage (PR) and/or disappearance (CR) after treatment. The secondary outcomes of this study were the identification of predictive factors for effective treatment and observation of the rate of post-procedural adverse events. We measured the absolute ethanol dose as the actual volume (ml) of ethanol used during the procedure, and calculated the relative ethanol dose by calculating the volume of ethanol per volume of tumor (ml/cm.^3^) based on the cube of the length of the longest axis. The definition and severity of adverse events that occurred after the procedure were evaluated according to the lexicon for endoscopic adverse events built by the American Society for Gastrointestinal Endoscopy [12].

### Statistical analysis

Continuous variables were provided as median values with range and were analyzed using Student’s *t* test. Categorical variables are presented as numbers and percentages and were analyzed using a Chi-squared test or Fisher’s exact test. Kaplan–Meier survival analysis and log-rank tests were used to compare the time to reach CR or objective response between subgroups as follows: (1) tumor type as PNET or SPT, (2) tumor size greater or less than 20 mm, and (3) higher or lower dose of ethanol usage. Multivariable Cox proportional hazards analysis was conducted using variables with a p value of less than 0.25 in a univariable analysis in order to find factors affecting response to treatment. Statistical significance was set at *p* < 0.05. Statistical analyses were performed using SPSS v.23.0 (IBM Corp., Armonk, NY, USA).

## Results

### Study population and baseline characteristics

**Fig. 1:**
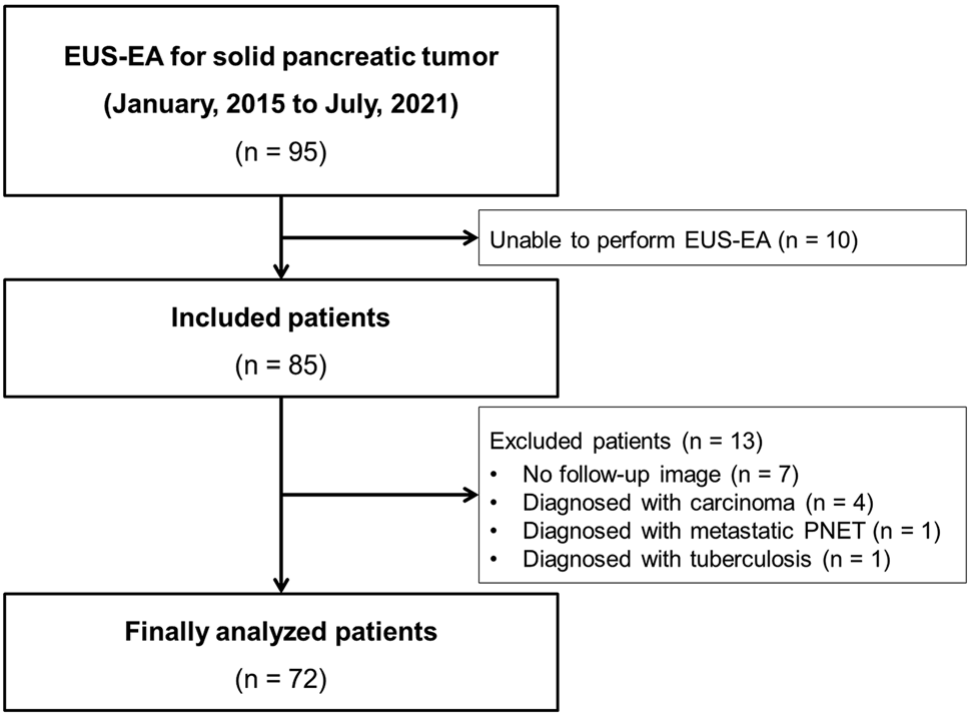
Flowchart of this study. EUS-EA, endoscopic ultrasound-guided ethanol ablation; PNET, pancreatic neuroendocrine tumor

95 patients who had been scheduled to undergo EUS-EA for solid pancreatic masses were retrospectively reviewed. Ten patients were unable to under go EUS-EA for various reasons, including lack of visibility of the pancreatic lesion via EUS and likeliness of the anatomical location of the tumor to cause fatal adverse events. Patients excluded from the study include 7 patients who did not undergo follow-up imaging after the procedure, 4 patients who were diagnosed with carcinoma by final pathology, 1 patient who was diagnosed with metastatic PNET after the procedure, and one patient who was diagnosed with pancreatic tuberculosis. Therefore, 72 patients were included in this study (Fig. 1). The baseline patient characteristics are shown in Table 1. The median age of the patients was 47.5 years (range 20–78 years) and 28 (38.89%) male patients were included in this study. Seven (9.7%) patients complained of symptoms related to PNETs. Approximately half of masses were located in the head or neck of the pancreas. A total of 40 (55.56%) patients were diagnosed with a non-functioning PNET, 7 (9.72%) with a functioning PNET, and 25 (34.72%) patients with an SPT. Among these patients, 61 (84.72%) were diagnosed with pathologic confirmation and the other 11 (15.28%) were diagnosed on the basis of clinical features and imaging studies. The median follow-up period was 26.0 months (range 3.2–149.7 months).

**Table 1:** Baseline characteristics of study patients

		*N* = 72
Age	Median (range), years	47.5 (20–78)
Sex	Male	28 (38.89%)
	Female	44 (61.11%)
Symptomatic	Yes	7 (9.72%)
	No	65 (90.28%)
Location	Head	33 (45.83%)
	Body	25 (34.72%)
	Tail	14 (19.44%)
Cystic degeneration or necrotic portion	Yes	7 (9.72%)
	No	65 (90.28%)
Diagnosis	Non-functioning PNET	40 (55.56%)
	Functioning PNET	7 (9.72%)
	SPT	25 (34.72%)
Pathologic confirmation	Yes	61 (84.72%)
	No	11 (15.28%)
Total follow-up period	Median (range), months	26.05 (3.23–149.77)

*PNET* pancreatic neuroendocrine tumor; *SPT* solid pseudopapillary tumor

### Result of the EUS-EA for solid pancreatic tumors

Detailed information and procedure results are presented in Table 2. The median initial tumor size was 1.5 cm (range 0.7–3.3 cm). The median volume of ethanol usage for each mass was 1.30 ml (range 0.1–13.0 ml), and the median ethanol volume per tumor volume was 0.35 ml/cm^3^ (range 0.004–3.207 ml/cm^3^). Most procedures were performed using a 22-gauge needle (93.1%). The median follow-up duration after the procedure was 18.4 months (range 0.6–62.5 months).

**Table 2:** Detailed information and result of the procedure

Total number of patients		72
Ablative size of tumor	Median size (range), cm	1.5 (0.7–3.3)
Ethanol dose	Median volume of ethanol used (range), ml	1.30 (0.1–13.0)
	Median ethanol per diameter (range), ml/cm^3^	0.356 (0.004–3.207)
Post-procedural follow-up period	Median duration after procedure (range), months	18.42 (0.63–62.50)
Needle	19G	1 (1.39%)
	22G	67 (93.1%)
	25G	4 (5.56%)
Final size of tumor	Median size (range), cm	1.2 (0–4.0)
Morphological response	CR	8 (11.1%)
	PNETs	6
	SPTs	2
	PR	40 (55.6%)
	PNETs	25
	SPTs	15
	SD	16 (22.2%)
	PNETs	12
	SPTs	4
	PD	8 (11.1%)
	PNETs	4
	SPTs	4
Clinical response	Clinical CR for functional PNET	7 (100%)
Surgical resection during study period		3 (4.2%)

*EUS*-*EA* endoscopic ultrasound guided ethanol ablation; *PNET* pancreatic neuroendocrine tumor; *SPT* solid pseudopapillary tumor; *CR* complete remission; *PR* partial remission; *SD* stable disease; *PD* progressive disease

The median tumor size in the final imaging at the end of the follow-up was 1.2 (range 0–4.0 cm). A total of 8 (11.1%) patients showed morphological CR, 40 (55.6%) showed PR, 16 (22.2%) showed SD, and 8 (11.1%) showed PD after the procedure. All 7 functioning PNETs showed clinical CR after the procedure. Three (4.2%) patients underwent surgical resection after the procedure.

### Predictive factors for morphological response

**Fig. 2:**
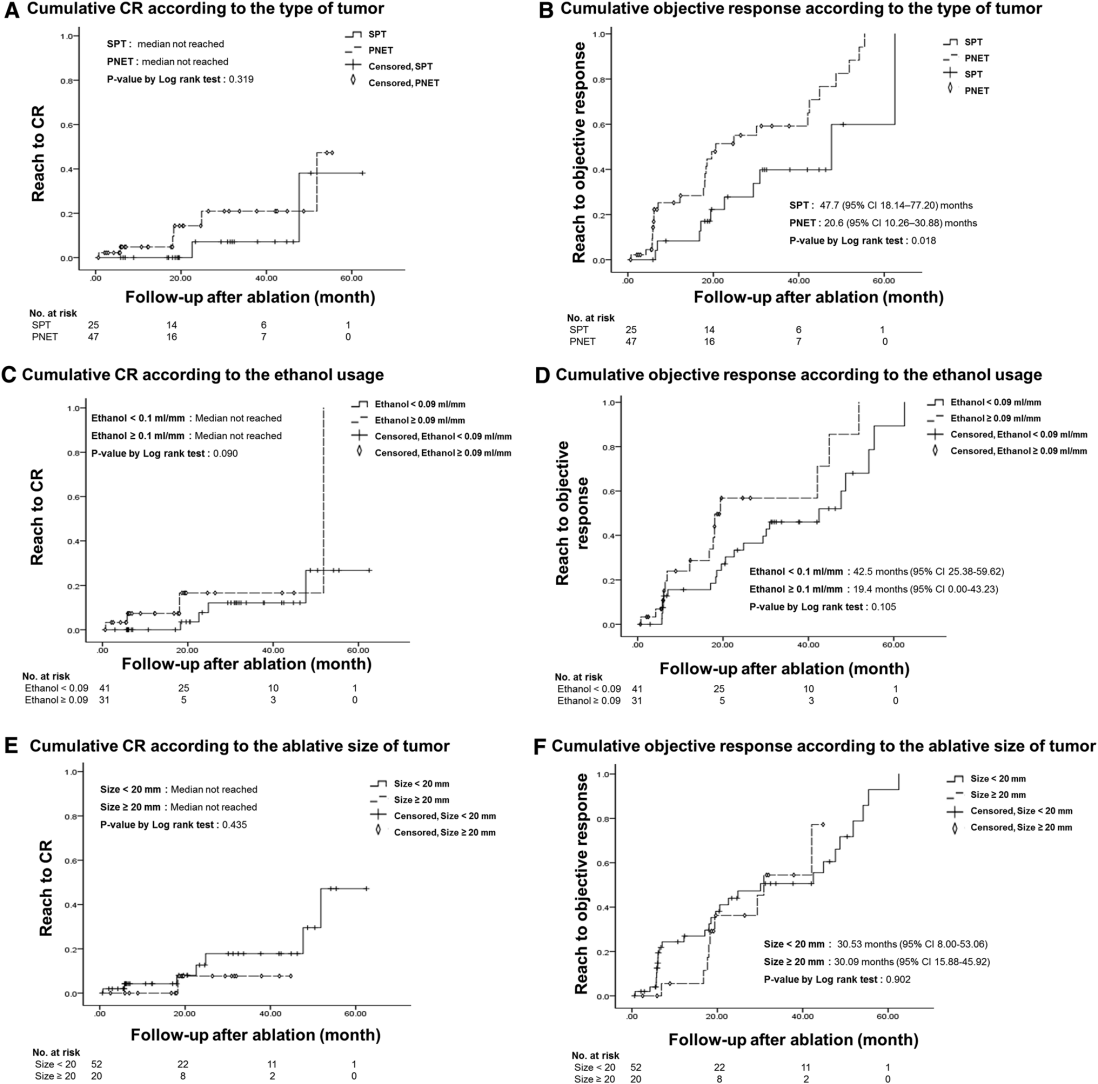
Cumulative completion remission or partial response according to key features. (**A**). Cumulative CR according to the type of tumor, (**B**). Cumulative response according to the type of tumor, (**C**). Cumulative CR according to the amount of ethanol used, (**D**). Cumulative response according to the amount of ethanol used, (**E**). Cumulative CR according to the ablative size of tumor, (**F**). Cumulative response according to to the ablative size of tumor. *CR*, complete remission; *OR*, objective response; *PNET*, pancreatic neuroendocrine tumor; *SPT*, solid pseudopapillary tumor

We attempted to identify features that could predict morphological CR. According to the result of Kaplan–Meier survival analysis, the type of tumor did not affect CR (Fig. 2A; median not reached in both groups; *p* = 0.319), but PNETs (Fig. 2B; median 20.6 months, 95% CI 10.26–30.88) showed significantly shorter duration to reach objective response than SPTs did (Fig. 2B; median 47.7 months, 95% CI 18.14–77.20; *p* = 0.018). Ethanol dose affected CR (Fig. 2C; median not reached in both group; *p* = 0.026) and objective response (Fig. 2D; for ethanol dose same or less than 0.35 ml/cm^3^: median 42.5 months; 95% CI 25.34–59.66; for ethanol dose higher than 0.35 ml/cm^3^: median 19.6 months; 95% CI 10.17–29.09; *p* = 0.006). The size of the tumor did not affect CR (Fig. 2E; median not reached in either group; *p* = 0.435) or objective response (Fig. 2F; for tumor size less than 2 cm: median 30.53 months; 95% CI 8.00–53.06; for tumor size same or greater than 2 cm: median 30.09 months; 95% CI 15.88–45.92; *p* = 0.902).

Table 3 shows the results of the Cox proportional hazard analysis on predictive factors for morphological response. The use of an ethanol dose higher than 0.35 ml/cm^3^ (Hazard ratio (HR) 4.75, 95% CI 1.08–20.92; *p* = 0.039) was shown to predict CR via univariable analysis with statistical significance, but there were no other statistically significant factors for predicting CR via univariable or multivariable analysis. In terms of objective response, univariable analysis revealed that PNETs (HR 2.42, 95% CI 1.13–5.20; *p* = 0.022) and an ethanol dose higher than 0.35 ml/cm^3^ (HR 2.55, 95% CI 1.27–5.12; *p* = 0.008) could predict objective response significantly better. With a multivariable analysis, PNETs still showed significantly higher predictive objective response (HR 3.34, 95% CI 1.07–10.43; *P* = 0.038), but the use of an ethanol dose higher than 0.35 ml/cm^3^ did not (HR 1.64, 95% CI 0.75–3.61; *p* = 0.215).

**Table 3:** Predictive factors for morphological response by Cox proportional hazard analysis

Factors	Predictive factors for CR				Predictive factors for objective response			
	Univariable		Multivariable		Univariable		Multivariable	
	HR (95% CI)	P	HR (95% CI)	P	HR (95% CI)	P	HR (95% CI)	P
Age ≥ 50 years	3.59 (0.71–18.24)	0.123	3.96 (0.55–28.32)	0.171	1.55 (0.79–3.02)	0.201	0.64 (0.25–1.60)	0.338
Female	0.82 (0.19–3.45)	0.787			0.85 (0.42–1.73)	0.661		
Head	6.62 (0.78–56.45)	0.084	9.82 (0.81–119.6)	0.073	1.55 (0.76–3.14)	0.229	1.87 (0.87–4.03)	0.109
Size ≥ 2 cm	0.44 (0.05–3.73)	0.448			0.96 (0.44–2.11)	0.922		
Presence of cystic portion	0.42 (0.00–3279.3)	0.581			0.91 (0.28–2.99)	0.874		
PNET	2.23 (0.44–11.20)	0.331			2.42 (1.13–5.20)	0.022	3.34 (1.07–10.43)	0.038
Use of ethanol dose more than 0.35 ml/cm^3^	4.75 (1.08–20.92)	0.039	2.27 (0.39–13.40)	0.364	2.55 (1.27–5.12)	0.008	1.64 (0.75–3.61)	0.215

*CR* complete remission; *CI* confidence interval; *HR* hazard ratio; *PNET* pancreatic neuroendocrine tumor

### Patients who underwent surgical resection

Among the 3 patients who underwent surgery, 2 decided to undergo surgery immediately after the pathologic confirmation, based on the recommendation of the attending physician who was concerned about the poor prognosis of grade 2 PNETs, while the other patient underwent surgical resection due to increased SPT size and newly found peri-pancreatic lymph node enlargement 6 months after EUS-EA. The surgical pathological stage of the resected SPT was pT3N0. Among the patients who underwent surgical resection, intratumoral ethanol-related necrosis was reported as 70–90%, including an SPT which showed a 20% size increase post-procedure. No patients experienced tumor recurrence after surgery (Table 4).

**Table 4:** Clinical and pathological information of patients who underwent surgical resection

	Patient 1	Patient 2	Patient 3
Age / Sex	28 / Female	37 / Male	73 / Female
Location	Head	Body	Tail
Reason for ablation	Patient’s wish	Patient’s wish	Patient’s wish
Reason for surgery	Diagnosis with grade 2 NET	Increased size of SPT after ablation and newly found peri-pancreatic lymph node enlargement	Diagnosis with grade 2 NET
Duration till surgery after ablation	72 days	189 days	71 days
Ablative / Final size	2.5 / 2.3 cm	3.3 / 4.0 cm	2.5 / 2.3 cm
Response	SD	PD	SD
Ethanol volume	0.166 ml/cm^3^	0.362 ml/cm^3^	0.421 ml/cm^3^
Adverse event after ablation	No	No	No
Surgery	PPPD	Distal pancreatectomy	Distal pancreatectomy
Histologic diagnosis	NET	SPT	NET
Ki-67	6.1%	1.3%	8.2%
Amount of ethanol related necrosis in entire resected tumor	70%	70%	90%
Lymph node involvement	No	No	No

*NET* neuroendocrine tumor; *SPT* solid pseudopapillary tumor; *SD* stable disease; *PD* progressive disease

### Adverse events after the procedure

A total of 23 (31.9%) patients experienced adverse events after EUS-EA for solid pancreatic masses (Table 5). During follow-up, there were no malignant transformation or metastatic cases. Post-procedural pancreatitis occurred in 11 (15.3%) patients, abdominal pain which was not caused by pancreatitis or perforation in 11 (15.3%) patients, pancreatic enzyme elevation without abdominal pain in 4 (5.6%) patients, and post-procedural duodenal stricture in 1 (1.4%) patient. Almost all adverse events showed mild to moderate severity (91.3%), but there were 2 (8.7%) severe cases. One patient underwent EUS-EA for a suspected 1.3 cm pancreatic body PNET and suffered from severe abdominal pain with fever after the procedure. CT revealed severe necrotizing pancreatitis due to ethanol injection. Eleven days after the procedure, symptoms improved with conservative management including antibiotics, and the patient was discharged. One month later, follow-up EUS revealed no evidence of necrosis. The other patient underwent EUS-EA for a suspected 1.2 cm pancreatic head PNET. The patient visited the emergency department complaining of severe abdominal pain and vomiting lasting 2 days. CT revealed a duodenal stricture, which was suspected to be due to a post-procedural peripancreatic edema. Symptoms improved after insertion of partially covered self-expandable duodenal metal stent to stricture site, which was removed after 1 month without any adverse events.

**Table 5:** Adverse events after the procedure

		*N* (%)
No adverse events		49 (68.1)
Adverse events	Total	23 (31.9)
	Post-procedural pancreatitis	11 (15.3)
	Abdominal pain (not caused by pancreatitis or perforation)	11 (15.3)
	Duodenal stricture	1 (1.4)
Severity	Severe	2 (8.7)
	Mild to moderate	21 (91.3)

## Discussion

In this retrospective study of patients diagnosed with a solid pancreatic tumor who were treated with EUS-EA, 11.1% showed morphological CR, and 66.7% reached objective response after median follow-up of 18.5 months post-procedure. Tumors that showed an objective response during the observation period did not progress or grow further. PNETs appeared to respond better to this procedure than SPTs. Taking clinical response into consideration, both functioning and non-functioning PNETs are good indications for this procedure. A total of 23 (31.9%) patients suffered from adverse events after the procedure. However, almost all patients improved with conservative management.

The morphological CR rate in this study (11.1%) was much lower than that of previous studies (60–75%) [7, 8, 13]. A recent study of 33 cases with small PNETs reported 45% CR after one session and 60% CR after two sessions of EUS-guided ethanol-lipiodol ablation during a very long-term follow-up period with a median of 42 months [8]. There is no gold standard for assessing responses of EUS-EA treatment for asymptomatic masses. Researchers in the aforementioned study defined CR as the absence of any area of enhancement contiguous with the ablated tumor in contrast-enhanced CT or contrast-enhanced EUS imaging and negative cytology upon repeat EUS-guided fine-needle biopsy at the 3-year follow-up [8]. These criteria seem to be very strict and to increase short-term medical expenses, but they allow a broader range of CR by distinguishing postoperative remnant scars from the actual viable portion of the tumor. In our institution, these criteria are difficult to apply because contrast-enhanced EUS only recently became available, and follow-up biopsies were not performed unless an inconclusive diagnosis was made. The objective response rate (65.8%) in this study was similar to those in previous studies. It is noteworthy that, in this study, ethanol-related tumor necrosis was observed in 70–90% of the area within the resected tumor. All functioning PNETs showed clinical CR, although only two cases reached morphological CR. In the future, it might be necessary to evaluate viability using reasonable tools such as contrast-enhanced EUS.

Pathology and ethanol dose (> 0.35 ml/cm.^3^) appeared to affect the treatment response. PNET was found to be a significant predictive factor, as confirmed by multivariable analysis. The excellent effectiveness of EUS-EA on functioning PNETs was in line with the results of previous studies [6–10, 13]. PNETs and SPTs of the pancreas differ significantly in many clinical and pathologic features, but it is difficult to explain the better response of PNET to ethanol ablative therapy. Some heterogeneous pathologic features of SPTs such as encapsulation, solid and cystic components, and peripheral calcification might affect the efficacy of EUS-EA by disturbing the homogenous distribution of ethanol [14, 15].

Based on this and previous studies, the proper indication of solid pancreatic masses for EUS-EA can be proposed as follows: patients who are not eligible for surgery or who are reluctant to surgery, small grade 1 PNETs (< 20 mm) rather than SPTs, and functional PNETs. Because symptomatic PNETs have surrogate symptoms to evaluate the effect of treatment, functional PNETs could be a proper indication for EUS-EA. For nonfunctional PNETs, grade 1 PNETs could be a proper indication for EUS-EA. PNETs of grade 2 or 3 have a relatively high probability of metastasis or lymph node involvement, even though they are smaller than 20 mm [16–19]. Unexpectedly, the size of the ablated tumor (≥ 20 mm) was not a factor that affected the outcome of the procedure in this study. However, 87.5% (seven of eight cases) of CR was achieved in PNETs smaller than 20 mm. PNETs smaller than 20 mm seemed to be a more appropriate indication for EUS-EA, considering the probability of advanced disease and overall prognosis [17, 19–21].

To the best of our knowledge, this study included the largest number of patients who underwent EUS-EA for solid pancreatic tumors, including PNETs and SPTs. Previous studies have shown the efficacy of this procedure, mainly for functioning PNETs. In this study, we attempted to establish proper indications and to optimize the procedure not only for functional PNETs but also for nonfunctional PNETs and SPTs. Most adverse events after the procedure were resolved with conservative management, which indicates that this procedure can be safely applied for extended indications in the future. In addition, treatment with EUS-EA may be a good alternative for patients who require of surgical resection over a wide area due to scattered PNETs within the pancreas, or for patients with an inherited syndrome that increases the likelihood of developing multiple PNETs [22–24].

This study had several limitations. First, this is a retrospective study with limited number of patients and there was no unified protocol which was established prior to the study. Second, it was difficult to evaluate whether ethanol was sufficiently injected into the entire tumor. Efforts to inject ethanol into every corner of the tumor and to observe the cloudy appearance of the entire tumor area are considered important. Previous studies have not quantitatively proposed the appropriate amount of ethanol to inject. This study showed that it is necessary to inject a sufficient amount of ethanol for the tumor volume. Third, it was difficult to accurately evaluate the response to ethanol ablation. If CT or EUS showed a non-enhancing area in the tumor after ethanol ablation, we could not differentiate whether the area was a scar change after tumor necrosis or whether it contained viable tumor cells.

In conclusion, EUS-EA for solid pancreatic neoplasms seems feasible as a local treatment modality for patients who refuse surgery or who have poor surgical performance. PNETs seem to be a better candidate for EUS-EA, and sufficient injection of ethanol is important to achieve a good response to the procedure. Determination of the proper indications for this procedure, the amount of ethanol to use, a post-operative follow-up protocol, and evaluation methods for response should be further pursued through larger prospective studies in the future.
